# Strong association between earlier abuse and revictimization in youth

**DOI:** 10.1186/1471-2458-14-715

**Published:** 2014-07-14

**Authors:** Helena Blom, Ulf Högberg, Niclas Olofsson, Ingela Danielsson

**Affiliations:** 1 Department of Clinical Sciences, Obstetrics and Gynaecology, Umeå University, SE-901 87 Umeå, Sweden; 2 Department of Obstetrics and Gynaecology, Sundsvall Hospital, Sundsvall, Sweden; 3 Department of Women’s and Children’s Health, Uppsala University, SE-751 85 Uppsala, Sweden; 4 Department of Research and Development, Västernorrland County Council, Sundsvall, Sweden; 5 Department of Medical and Health Sciences, Division of Community Medicine, Social Medicine and Public Health Science, Linköping University, Linköping, Sweden

**Keywords:** Youth, Adolescents, Abuse, Violence, Cross-sectional study, Sociodemographics, Risk factors, Revictimization

## Abstract

**Background:**

Violence victimization among youth is recognized as a public health problem. The objective was to analyze the risk pattern of emotional, physical, and sexual abuse during the past 12 months by gender, sociodemographic factors, health risk behaviors, and exposure to abuse before the age of 15, among young men and women attending youth health centers in Sweden.

**Methods:**

A cross-sectional survey was conducted using a nationally representative sample of youth health centers. A total of 2,250 young women and 920 young men aged 15–23 completed a self-administered questionnaire. Odds ratios (OR) and adjusted odds ratios (AOR) with 95% CI were calculated.

**Results:**

A consistent and strong association was noted between exposure to all types of violence during the past year and victimization before the age of 15 for all types of violence for both women and men. The only exceptions were childhood sexual victimization and sexual violence during the past year for men. Younger age was associated with all violence exposure for the women and with emotional violence for the men. For the women, drug use was associated with all types of violence, while the association with hazardous alcohol use and not living with parents was restricted to physical and sexual violence exposure, present smoking was restricted to emotional and physical violence exposure, and partnership and living in urban areas were restricted to sexual violence. For men, not being partnered, hazardous alcohol consumption, and drug use meant increased risk for physical violence, while smoking and living in urban areas were associated with sexual violence. After adjustment, immigration had no association with violence exposure.

**Conclusions:**

Violence victimization in young men and women is often not a single experience. Findings underline the importance of early interventions among previously abused youth.

## Background

Youth violence is recognized as a significant public health problem [1] and the strong associations between violence exposure and health risk behaviours and physical and psychological ill health are well documented [1-3].The WHO categorizes violence victimization into self-inflicted, interpersonal, and community violence [4]. Interpersonal violence victimization in adolescents and young adults is high and the prevalence varies according to type of violence and perpetrator [1,4-9] with prevalence rates for physical violence victimization during the past year varying from 18-20% for women and 25-27% for men [5,10]. Dating violence, often described as physical and sometimes sexual violence in a dating relationship, is most often used in American literature [8,9] while in other reports, the perpetrator is not the focus, only the violence exposure per se [5,7]. To understand risk factors for interpersonal violence victimization, different explanatory levels, including societal, community, relationship and individual factors, may be addressed [4,6]. The examination of risk factors for violence victimization may provide an opportunity to identify individuals with possible risk factors eligible for prevention strategies.

Young men have a higher prevalence of physical violence exposure than young women [4,5,10], while experience of sexual abuse consistently shows a higher prevalence in young women [5,11,12]. It is well-known that low age is a risk factor for exposure to violence in both young men and women [6,10,13], although in adolescence and young adulthood, it is unclear at which age the violence exposure declines. A longitudinal U.S. study on high school students identified living in a single-parent household as predictive for physical violence victimization in young women but not in young men [14].

The American experience is that parents’ education and socioeconomic status may be more strongly associated with violence exposure than ethnicity [15]. One U.S. study suggests immigration status to be protective for dating violence among adolescent girls [16]. The association between violence victimization among youth and immigration status in a European context is not known.

Individual risk factors for violence victimization may include health risk behaviors and negative life experiences. Hazardous alcohol use or binge drinking are sometimes found to be associated with violence exposure in youths [8,17], but not always [2,12,18,19]. Smoking and drug use are more constantly associated with violence victimization [2,8,19]. It has been shown that people with childhood victimization are more likely to take part in high-risk behaviours [20,21]. Although numerous studies have identified exposure to childhood physical and/or sexual abuse as significantly increasing the risk for later violence exposure in adult women [7,22], sometimes including adult men [23,24] and young adults [18,25,26], little attention has been paid to exposure to emotional abuse during childhood and later abuse.

WHO applies the public health perspective in the ecological model as a theoretical approach to understand causes of interpersonal violence. This theory includes different explanatory levels a) societal, b) community, c) relationship- and d) individual factors [4,6]. Societal factors can be social and cultural norms regarding gender roles, endorsement of violence as a normal method to resolve conflicts and honour-based-violence. The community context represents institutions and social structures – such as attitudes in peer groups, neighbourhood and poverty. Relationship can include family factors such as witnessing domestic violence, parental substance abuse and parental mental illness. Individual level identifies biological and personal factors for each individual including previous trauma and substance abuse.

In criminology research the current explanatory hypotheses on the links between victimization and revictimization are the population heterogeneity hypothesis, that focus on stable individual characteristics that make the person more accessible to violence , and the state dependent hypothesis, that focus on changes in behaviour after victimization [27].

In an earlier study, we showed that young men and women visiting youth health centers in Sweden have a high and gendered exposure to violence. Young women were more exposed to emotional and sexual violence, while young men were more exposed to physical violence. The overlap between emotional, sexual, and physical abuse was considerable, specifically concerning sexual abuse [5].

In studies on childhood and young adolescence (age 2 to 17 years) the poly-victimization (experience of victimization in several contexts e.g. sexual victimization, maltreatment, property crime, physical assault, peer/sibling victimization and witnessed/indirect victimization), indicates an elevated risk for additional victimization [28]. The different pathways for being a poly-victim include residing in a dangerous community, living in a dangerous family, having a multiproblem family environment or having emotional problems that increase risk behaviour [29]. The research on victimization in childhood indicates that victimization may, in some children, be more seen as condition with multiple victimization, rather than an isolated event [20].

The objective of this study was to analyze the risk pattern of exposure to emotional, physical, or sexual abuse during the past 12 months by gender, sociodemographic factors, health risk behaviors, and exposure to violence before the age of 15, among young men and women attending youth health centers in Sweden.

## Methods

### Settings

A nationally representative convenience sample of nine youth health centers in Sweden participated in a cross-sectional study from February until June 2007. Most cities in Sweden have youth health centers, where young people from ages 13 to 23 are eligible for contraceptive advice and advice on gynecological problems, social, psychological, or physical problems, and ill health. From the Swedish National Health Survey for the years 2006–08, it was reported that 25% of young women aged 16–25 and 5% of corresponding young men had attended a youth health center during the past three months [30].

The nine centers, which were located in urban and rural areas in both the northern and southern parts of the country and the three biggest cities in Sweden, consecutively recruited women and men aged 15–23, who all answered a questionnaire. Each center recruited according to their annual number of visits. The external dropout rate was 12% among the young men and 14% among the young women [5]. Detailed information about the study, description of the external dropout, methodological considerations, and part of the questionnaire are provided elsewhere [5]. Ethical aspects are presented in a separate section in this paper. None of the participating youth health centers had a specific profile concerning violence or abuse.

The internal dropout rate for the questions according age, any drug use, hazardous alcohol consumption, daily smoking, immigrant status, family structure, and having a current partner varied between 1 and 3% for the young women and 3 and 5% for the young men. For emotional, physical, and sexual abuse, the internal dropout rate varied from 3-4% for the young women and 6-9% for the young men. We used violence and abuse synonymously [5].

### Measures

#### Sociodemographics

Different sociodemographic variables from the questionnaire were assessed, including gender, age (as a continuous variable), place of living, family structure, immigrant status, and having a partner. Place of living was dichotomized according to number of inhabitants of the city of each youth health center into big cities with more than 300,000 inhabitants and small cities with less than 300,000. Family structure was stratified into living with (a) both biological parents, (b) one biological parent, and (c) some other arrangement (e.g., with friend(s), girl- or boyfriend, alone). Immigrant status was dichotomized into immigrants (foreign-born youth and Swedish-born youth with two foreign-born parents) and youth with Swedish-born parents. The answer to the question “Do you have a current partner?” was dichotomized into yes or no. Sociodemographic variables were based on questions from validated surveys, i.e., the Swedish National Public Health Survey and “Q90,” a health survey aimed at adolescents [31,32]. No socioeconomic information about the parents was asked for in the questionnaire.

#### Emotional, physical, and sexual abuse

The questions on abuse were taken from the validated NorVold Abuse Questionnaire [33,34] with detailed and specific questions about experiences of emotional, physical, and sexual abuse, ranging from what is called mild to moderate and severe abuse. As an example, severe emotional abuse is described as: “Have you experienced living in fear because someone repeatedly and for a long period has threatened you or somebody close to you?” and moderate physical abuse as: “Have you experienced anybody hitting you with his/her fist(s) or with a hard object, kicking you, pushing you violently, giving you a beating, or doing anything similar to you?”. The time spans involved were violence exposure before 15 years of age and during the past 12 months. Detailed information and part of the questionnaire are provided elsewhere [5]. In this study, moderate and severe violence exposure of emotional, physical, and sexual abuse before the age of 15 were assessed as risk factors/independent variables. The dependent variables in the analyses were : 1) exposure to any (mild, moderate, severe) emotional abuse during the past 12 months/past year, 2) exposure to any physical abuse during the past 12 months, and 3) those exposed to any sexual abuse during the past 12 months.

Young people abused to more than one type of violence (emotional, physical and/or sexual abuse) during the past 12 months were defined as multi-abused, and this variable was also analyzed as a dependent variable.

#### Alcohol, drugs and smoking

AUDIT-C, the three first questions in the WHO’s test called AUDIT (Alcohol Use Disorders Identification Test), was used to identify hazardous alcohol consumption [35]. AUDIT-C is regarded to be equal to, or even better than, AUDIT for identifying hazardous consumption in both adults and adolescents [36]. The three first questions including 1) how often alcohol is consumed, 2) how many glasses consumed on a normal occasion, and 3) how often a fairly large amount are drunk on the same occasion, giving an index scoring from 0–12. In this study the cut-off values of ≥5 for young women and ≥6 for young men were applied for hazardous drinking patterns [36,37]. Any use of drugs (e.g., hash, marijuana, ecstasy, GHB, anabolic steroids) during the past 12 months was dichotomized into no or yes. Daily smoking was dichotomized into no or yes. The questions on drug abuse and daily smoking were taken from the Swedish National Public Health Survey [32].

### Statistical methods

Descriptive statistics were analyzed for the total sample. Student’s T-test was used to analyze differences in numerical values. Fischer’s exact test was used in small samples. All analyses were stratified by gender. Pearson’s Chi^2^ test was used for differences in frequencies. Comparison was made between those exposed and those not exposed to violence during the past 12 months.

The first steps of the logistic analyses were used to examine the univariate associations between possible sociodemographic and individual risk factors and exposure to violence during the past 12 months. The sociodemographic factors were age, place of living, family structure, having a partner, and immigration status, while the individual risk factors were hazardous alcohol consumption, daily smoking, drug use, and exposure to moderate/severe emotional, physical, or sexual violence before the age of 15. Multivariate logistic regression models were constructed to examine the associations between all sociodemographic factors and individual risk factors, including abuse before the age of 15, and violence exposure during the past 12 months. A theory driven regression approach was used, and factors were added to the model in stages. At each stage, an additional factor was added or removed to reach the best fitting model. All socio-demographic and individual risk factors proved to be significant for one or several dependent variables, and thus all where included in the model. The same model was used in both men and women. Crude and adjusted odds ratios were estimated. A p-value of <0.05 was considered statistically significant. All statistical analyses were performed by SPSS (version 19 and 20).

### Ethics

The Regional Ethical Review Board in Umeå approved the study (Dnr 06-118 M). The participants were informed verbally and in writing about the study by the midwife/social worker and oral informed consent given to the same person was considered sufficient. In Sweden the position of the Central Ethical Review Board is that consent from parents/guardians is not needed for youths 15 years and older, if the person is judged to understand the information and able to make a self-governed decision. All staff was thoroughly informed about the ethical standpoints and adolescents with severe medical or psychological disease or mental retardation were excluded from participating in the study [5].

## Results

In all, 2,250 women and 920 men answered the questionnaire. Descriptive characteristics are presented in Table 1. The men were a little older in general, while 14.5% and 9.3% of the women and men, respectively, were 15 years old. Women were more often partnered and had more often been exposed to sexual violence before the age of 15 than men, while men more often were immigrants and had a hazardous alcohol consumption and drug use. The prevalence of the independent variables for men and women exposed and non-exposed to violence during the past year are presented in Table 2. Young women exposed to emotional, physical, and sexual violence during the past 12 months were significantly more often living with one biological parent, had hazardous alcohol consumption, used drugs, were present smokers, and had experienced violence before the age of 15 than non-exposed. Young men exposed to emotional, physical, and sexual violence during the past year were significantly more often present smokers and had experienced violence before the age of 15 than non-exposed. Hazardous alcohol consumption and drug use were significantly more prevalent among men exposed to physical abuse past year than non-exposed.

**Table 1:** Descriptive characteristics of the young women and men recruited to the study

	**Young women N = 2250**	**Young men N = 920**
Age years mean (lowest-highest)	17.9 (15–23)	18.9 (15–23)***
Place of living		
Small cities (%)	39	36
Big cities (%)	61	65
Living with		
Both parents (%)	39	37
One biological parent (%)	33	29
Someone else (%)	28	34**
Immigrants (%)	15	19**
Current partner (%)	60***	44
Hazardous alcohol consumption (%)	48	60***
Daily smoking (%)	27	24
Drug use (%)	15	30***
Exposed to violence <15 years		
Moderate-severe emotional (%)	24	24
Moderate-severe physical (%)	15	27***
Moderate-severe sexual (%)	9***	2

**p < 0.01, ***p < 0.001 for the difference between men and women.

**Table 2:** The prevalence of risk factors in women and men exposed (Exp) and non-exposed (NE) to emotional, physical, and sexual abuse during the past 12 months

	**Emotional abuse past year**				**Physical abuse past year**				**Sexual abuse past year**			
	**Women**		**Men**		**Women**		**Men**		**Women**		**Men**	
	**Exp**	**Exp/NE**	**Exp**	**Exp/NE**	**Exp**	**Exp/NE**	**Exp**	**Exp/NE**	**Exp**	**Exp/NE**	**Exp**	**Exp/NE**
	**N = 733/1517**		**N = 167/753**		**N = 406/1844**		**N = 251/669**		**N = 312/1938**		**N = 43/877**	
	**n**	**%**	**n**	**%**	**n**	**%**	**n**	**%**	**n**	**%**	**n**	**%**
Living in												
Small cities	264	36/40	55	33/36	142	35/39	84	34/36	95	30/40	9	21/36
Big cities	469	64/60	112	67/64	264	65/61	167	67/64	217	70***/60	34	79*/64
Living with												
Both parents	279	39/40	58	35/37	136	34/41	89	36/37	105	34/40	17	40/37
One biological parent	269	37**/30	53	32/29	163	41***/31	82	33/28	120	39*/32	17	40/29
Someone else	175	24/30	53	32/34	101	25/29	76	31/35	80	26/28	9	21/35
Immigrant	117	16/14	35	22/18	70	18/14	44	18/19	42	14/15	7	18/19
Current partner	409	57*/63	81	49/43	228	57/61	119	48/42	157	51***/62	17	40/44
Hazardous alcohol consumption	380	52*/46	93	56/61	246	61***/45	183	73***/55	183	59***46	27	63/60
Daily smoking	259	36***/22	50	31*/22	165	41***/23	77	31**/21	111	36***/25	20	48***/23
Drug use	152	21***/12	58	35/28	112	28***/12	105	42***/25	80	26***/13	14	35/29
Exposed to violence <15 y												
Moderate/severe emotional	303	41***/16	87	52***/18	174	43***/20	89	36***/20	123	39***/22	19	44**/23
Moderate/severe physical	183	25***/11	78	47***/23	135	33***/12	106	42***/21	85	27***/14	16	37/27
Moderate/severe sexual	119	16***/5	5	3/1.6	84	21***/6.3	7	2.8/1.5	65	21***/7	4	9.3/1.5

*p < 0.05, **p < 0.01, ***p < 0.001 for the differences between women and men exposed and non-exposed to violence.

The association between age and all types of emotional, physical, and sexual abuse declined with age for women; in men only for emotional violence (Tables 3, 4 and 5).

**Table 3:** COR and AOR (95% CI) for possible risk factors for young women (n = 733) and men (n = 167) exposed to emotional abuse during the past 12 months adjusted for sociodemographic and individual risk factors

	**Young women**				**Young men**			
	**Crude**		**Adjusted**		**Crude**		**Adjusted**	
	**OR**	**95% CI**	**OR**	**95% CI**	**OR**	**95% CI**	**OR**	**95% CI**
Age	**0.8**	**(0.8-0.9)**	**0.8**	**(0.8-0.9)**	0.9	(0.8-1.0)	**0.8**	**(0.8-0.9)**
Place of living								
Small cities	1		1		1		1	
Big cities	**1.2**	**(1.0-1.4)**	1.1	(0.9-1.4)	1.2	(0.8-1.6)	1.3	(0.9-2.0)
Living with								
Both parents	1		1		1		1	
One biological parent	**1.2**	**(1.0-1.6)**	1	(0.8-1.2)	1.2	(0.8-1.8)	0.9	(0.6-1.5)
Someone else	0.8	(0.7-1.1)	1	(0.8-1.3)	1	(0.7-1.5)	1.2	(0.7-2.0)
Swedish background	1		1		1		1	
Immigrant	1.2	(0.9-1.5)	1.1	(0.8-2.0)	1.3	(0.8-2.0)	1.1	(0.7-1.8)
Current partner								
Yes	1		1		1		1	
No	**1.3**	**(1.1-1.5)**	0.8	(0.6-1.0)	0.8	(0.5-1.1)	1.2	(0.8-1.8)
Hazardous alcohol consumption								
No	1		1		1		1	
Yes	**1.3**	**(1.1-1.5)**	1.2	(0.9-1.4)	0.8	(0.6-1.1)	0.9	(0.6-1.4)
Daily smoking								
No	1		1		1		1	
Yes	**1.9**	**(1.6-2.3)**	**1.4**	**(1.1-1.7)**	**1.5**	**(1.0-2.2)**	1.1	(0.7-1.8)
Use of any drug								
No	1		1		1		1	
Yes	**1.9**	**(1.5-2.4)**	**1.5**	**(1.1-2.0)**	**1.4**	**(1.0-2.0)**	1.2	(0.8-1.8)
Exposed to violence								
Emotional <15 y no	1		1		1		1	
Yes	**3.8**	**(3.1-4.6)**	**2.8**	**(2.2-3.6)**	**4.9**	**(3.4-7.0)**	**3.5**	**(2.3-5.3)**
Physical <15 y no	1		1		1		1	
Yes	**2.8**	**(2.2-3.5)**	**1.4**	**(1.1-1.9)**	**3**	**(2.1-4.3)**	**1.9**	**(1.3-2.8)**
Sexual <15 y no	1		1		1		1	
Yes	**3.4**	**(2.5-4.6)**	**2.1**	**(1.5-2.9)**	1.9	(0.7-5.5)	0.8	(0.2-3.4)

OR = odds ratios. CI = confidence interval. Age is a continuous variable. Significantly raised OR are in bold print.

**Table 4:** COR and AOR (95% CI) for possible risk factors for young women (n = 406) and men (n = 251) exposed to physical abuse during the past 12 months adjusted for sociodemographic and individual risk factors

	**Young women**				**Young men**			
	**Crude**		**Adjusted**		**Crude**		**Adjusted**	
	**OR**	**95% CI**	**OR**	**95% CI**	**OR**	**95% CI**	**OR**	**95% CI**
Age	**0.8**	**(0.8-0.9)**	**0.8**	**(0.7-0.9)**	0.9	(0.8-1.0)	0.9	(0.8-1.0)
Living in								
Small cities	1		1		1		1	
Big cities	**1.2**	**(1.0-1.5)**	1.1	(0.9-1.4)	1.1	(0.8-1.5)	1.0	(0.7-1.5)
Living with								
Both parents	1		1		1		1	
One biological parent	**1.6**	**(1.2-2.0)**	1.2	(0.9-1.6)	1.2	(0.9-1.7)	1.0	(0.7-1.5)
Someone else	1.1	(0.8-1.4)	**1.4**	**(1.0-1.9)**	0.9	(0.6-1.3)	1.0	(0.6-1.5)
Swedish background	1		1		1		1	
Immigrant	**1.3**	**(1.0-1.8)**	1.2	(0.9-1.7)	1.0	(0.7-1.4)	1.0	(0.6-1.6)
Current partner								
Yes	1		1		1		1	
No	1.2	(1.0-1.5)	0.9	(0.7-1.2)	0.8	(0.6-1.1)	**1.4**	**(1.0-1.9)**
Hazardous alcohol consumption								
No	1		1		1		1	
Yes	**1.9**	**(1.5-2.4)**	**1.7**	**(1.3-2.3)**	**2.2**	**(1.6-2.0)**	**2.3**	**(1.6-3.3)**
Daily smoking								
No	1		1		1		1	
Yes	**2.2**	**(1.8-2.8)**	**1.3**	**(1.0-1.7)**	**1.7**	**(1.2-2.4)**	1.3	(0.9-1.9)
Use of any drug								
No	1		1		1		1	
Yes	**2.8**	**(2.1-3.6)**	**1.9**	**(1.4-2.6)**	**2.3**	**(1.7-3.1)**	**2.0**	**(1.4-2.9)**
Exposed to violence								
Emotional <15 y no	1		1		1		1	
Yes	**3.0**	**(2.4-3.8)**	**1.8**	**(1.4-2.4)**	**2.2**	**(1.6-3.0)**	**1.6**	**(1.1-2.4)**
Physical <15 y no	1		1		1		1	
Yes	**3.8**	**(3.0-4.9)**	**2.3**	**(1.7-3.1)**	**2.7**	**(2.0-3.7)**	**2.3**	**(1.6-3.3)**
Sexual <15 y no	1		1		1		1	
Yes	**3.9**	**(2.8-5.2)**	**1.9**	**(1.3-2.8)**	1.9	(0.7-5.0)	1.5	(0.4-5.4)

OR = odds ratios. CI = confidence interval. Age is a continuous variable. Significantly raised OR are in bold print.

**Table 5:** COR and AOR (95% CI) for possible risk factors for young women (n = 312) and men (n = 43) exposed to sexual abuse during the past 12 months adjusted for sociodemographic and individual risk factors

	**Young women**				**Young men**			
	**Crude**		**Adjusted**		**Crude**		**Adjusted**	
	**OR**	**95% CI**	**OR**	**95% CI**	**OR**	**95% CI**	**OR**	**95% CI**
Age	**0.8**	**(0.8-0.9)**	**0.8**	**(0.7-0.9)**	**0.8**	**(0.7-0.9)**	0.8	(0.7-1.0)
Living in								
Small cities	1		1		1		1	
Big cities	**1.5**	**(1.2-2.0)**	**1.5**	**(1.2-2.1)**	**2.2**	**(1.0-4.5)**	**3.6**	**(1.4-9.2)**
Living with								
Both parents	1		1		1		1	
One biological parent	**1.5**	**(1.1-1.9)**	1.2	(0.9-1.7)	1.3	(0.6-2.5)	1.0	(0.5-2.2)
Someone else	1.1	(0.8-1.5)	**1.7**	**(1.2-2.5)**	0.6	(0.3-1.3)	0.7	(0.2-1.9)
Swedish background	1		1		1		1	
Immigrant	0.9	(0.7-1.3)	0.9	(0.6-1.3)	1.0	(0.4-2.3)	0.6	(0.2-1.5)
Current partner								
Yes	1		1		1		1	
No	**1.6**	**(1.2-2.0)**	**0.7**	**(0.5-0.9)**	1.2	(0.6-2.3)	0.7	(0.3-1.4)
Hazardous alcohol consumption								
No	1		1		1		1	
Yes	**1.7**	**(1.3-2.2)**	**1.4**	**(1.1-1.9)**	1.1	(0.6-2.1)	0.9	(0.4-1.9)
Daily smoking								
No	1		1		1		1	
Yes	**1.7**	**(1.3-2.1)**	1.0	(0.7-1.3)	**3.1**	**(1.7-5.9)**	**2.8**	**(1.3-5.9)**
Use of any drug								
No	1		1		1		1	
Yes	**2.3**	**(1.7-3.0)**	**1.6**	**(1.1-2.2)**	1.3	(0.7-2.5)	1.0	(0.4-2.1)
Exposed to violence								
Emotional <15 y no	1		1		1		1	
Yes	**2.4**	**(1.8-3.0)**	**1.7**	**(1.2-2.2)**	**2.6**	**(1.4-4.8)**	2.1	(0.9-4.5)
Physical <15 y no	1		1		1		1	
Yes	**2.4**	**(1.2-3.2)**	**1.4**	**(1.0-1.9)**	1.7	(0.9-3.1)	1.1	(0.5-2.5)
Sexual <15 y no	1		1		1		1	
Yes	**3.5**	**(2.5-4.8)**	**2.3**	**(1.6-3.4)**	**6.8**	**(2.1-22)**	2.8	(0.5-15)

OR = odds ratios. CI = confidence interval. Age is a continuous variable. Significantly raised OR are in bold print.

A consistent and strong association was noted between exposure to violence during the past 12 months and victimization before the age of 15 for both women and men (Tables 3, 4 and 5). For the women, the adjusted odds ratios for exposure <15 to emotional, physical, or sexual violence and exposure past year were 1.4-2.8, 1.8-2.3, and 1.4-2.3, respectively. For the men, the adjusted odds ratios for exposure <15 to emotional or physical violence and exposure past year were 1.9-3.5 and 1.6-2.3, respectively, while exposure to sexual violence <15 was not associated with abuse past year (Tables 2, 3, 4 and 5).

In women exposed to emotional violence during the past 12 months, there was also an association to smoking and drug use (Table 3). A similar risk profile was found in women who had been subjected to physical violence during the past 12 months, but there was also an association with hazardous alcohol consumption (Table 4) and not living with parents. For young women with experience of sexual violence, there were associations to hazardous alcohol use, drug use, not living with parents, partnered, and living in big cities with more than 300,000 inhabitants) (Table 5).

In men, besides childhood experience of violence, physical abuse past year was associated with not being partnered, hazardous alcohol consumption, and drug use (Table 4), while smoking and living in big cities showed increased risk for sexual violence (Table 5). Immigration status had no association with exposure to violence past year after adjustment for all other risk factors.

The odd ratios for the young women (n = 393) and men (n = 114) exposed to more than one type of violence during the past 12 months were calculated. A similar risk profile for multi-abused as for single-abused young women was seen with raised AORs for younger age, exposure to violence before the age of 15, and drug use. In the men exposed to more than one type of violence, the only raised AOR was for exposure to emotional violence before the age of 15.

## Discussion

There was a strong and consistent association between exposure to different forms of abuse during the past 12 months and exposure to violence before the age of 15 in both men and women. Lower age was a risk factor for all abuse in the young women; in men only for emotional abuse. Only a small number of associations with sociodemographic characteristics were noted, with the exception of sexual abuse and living in big cities for both young men and women. Immigrant status did not show any associations with violence exposure during the past 12 months. As for health risk behaviors, hazardous alcohol use was associated with physical and sexual abuse in women; in men, only with physical abuse. Smoking was moderately associated with emotional and physical abuse in women; in men, only with sexual abuse. Drug use was moderately associated with all violence exposures among women, but for men, only with physical abuse.

Having experienced emotional, physical, or sexual violence before the age of 15 was highly associated with abuse during the past 12 months, regardless of type, even though the odds ratios were highest for the same type of violence. In the young men, there was no significant association with exposure to sexual violence before the age of 15, regardless of type of abuse during the past 12 months. Since only few young men were exposed to sexual violence, the interpretation must be cautious [5]. Several studies have demonstrated a strong association between violence victimization as a child and violence exposure among adult women and young adult women and men [7,19,38-40]. Most often, the studied violence exposure, both as an adult/young adult and as a child, has been restricted to sexual violence, but sometimes physical violence is included [7,39]. In our study, sexual, physical, and emotional violence are included for both periods. Also, in our study, the violence exposure for the young adults is controlled for sociodemographic factors and health risk behaviors.

In this study we have used violence exposure before the age of 15 to describe childhood victimization [26]. The cut-off ages for childhood victimization varies in other studies, from prior to the initiation of dating [24] to being <18 years [22,23], being <14 [40], being 16 years [7] and sometimes no specific age limit [38]. In our study, neither the study design nor the questionnaire were devised to answer the question of when the violence before the age of 15 occurred. Consequently, it is not possible to know whether the violence was distant or close in time in the men and women exposed to violence during the past year. Some of the experiences of violence in the young men and women might be a continuum from before and after 15 years. This possibility might be one reason for the consistently strong association found between exposure to violence during the past 12 months and exposure before 15 years of age. It is also probable that some young men and women may have been repeatedly victimized, or even poly-victimized according to Finkelhor, but this was neither possible to analyze.[20,29].

In a previous article on the same material, we accounted for perpetrators of the violence among the adolescents’ violence exposure during the past 12 months [5]. Parents contributed to a small proportion of the perpetrators, while peers, strangers, and present or former partners were more often stated.

The strong association between earlier abuse and later violence exposure, found in our study, are in line with the literature [7,23,25,26,28,38-40]. The complexity of victimization in childhood and young adolescence [11,20], implicates the importance of addressing a broad spectra on violence exposures. By taking into account different types of abuse in a study population including adolescents and young adults, this study adds insights to the importance of not being restricted to an isolated violence exposure [20,21].

A strong association between emotional violence before the age of 15 and principally all types of abuse among the young men and women was found. Exposure to emotional violence before the age of 15 had strong associations in the multi-bused men compared to single-exposed men. There are only a few studies on earlier emotional abuse as a risk factor. In an American prospective study on college women, verbal abuse by parents was a significant predictor for adolescent dating violence [40].

The association between hazardous alcohol consumption/drug use and physical/sexual abuse in the young women found in this study is consistent with one study [41], although another did not find strong relationships [18]. The association between violence exposure and alcohol is complex. One population-based longitudinal study found that accumulation of violence exposures had an negative impact on adolescent alcohol use three years after the abuse [42] Another population based longitudinal study found strong links between victimization and alcohol/drug use, with gendered differences regarding bi- or uni-directional relationship [43].

Cigarette smoking has been studied as one of several risk behaviors in some studies and is most often found to be associated with violence in both young men and women [2,12], although after adjusting for other risk factors, the association in one study of high school students remained only in the young women [12]. In our study, daily smoking was weakly correlated with emotional and physical abuse in young women and strongly with sexual abuse in young men. Low economic status of the family has been demonstrated to be associated with higher exposure in youth violence in some studies [4,44]. In our study, no variable for socioeconomic status was available. Smoking could be regarded as a proxy for low socioeconomic status [45], thus indicating a relationship between violence exposure and low socioeconomic status.

Few clearly gendered differences were seen in the association between exposure to any violence before the age of 15 and abuse during the past 12 months, except for sexual violence. This data must be interpreted with caution, due to the few young men exposed to sexual violence [5]. In both genders, hazardous alcohol consumption, daily smoking, and drug use were associated with exposure to physical violence, although daily smoking was not significant in the young men. The association in the young women between drug use/daily smoking and sexual abuse during the past 12 months is consistent with one study [19] but inconsistent with another [12].

The major strengths of our study are the validated questionnaire on abuse, the national setup, the high participation rate in combination with a low internal dropout rate, and the fact that both young men and women were included. In many studies, only women are studied [6,7]. In addition, we addressed the complexity of violence victimization by including different types of violence exposure.

There are several weaknesses to our study. First, the study was cross-sectional, so conclusions about causality cannot be made. Second, we had no variable for environmental risk factors, including parents’ education, socioeconomic status, substance abuse and health, or other maltreatments during childhood besides abuse. Also, we decided not to control for the young people’s education, since the variable encoding for enrollment in a vocational or theoretical program in upper secondary school was found not to be reliable.

In many youth health centers in Sweden, questions about health-risk behaviors, including hazardous alcohol consumption and use of tobacco and drugs, have been routinely included for several years. In addition, screening for intimate partner violence during maternity care has become routine in Sweden. The National Center for Knowledge on Men’s Violence Against Women put forth in 2010 that there is strong evidence that all women seeking medical care should be routinely asked about earlier experience of violence [46]. Still, today there is no consensus for asking routine questions about abuse in the health care system in Sweden. An U.S. review recommends adolescent health care providers to screening efforts [1]. A meta-analysis on screening for intimate partner violence in healthcare settings indicates that screening does not seem to cause harm in the short term, although the evidence for improved outcome after screening was still considered insufficient [47]. On the basis of our study, we find it essential to discuss the use of questions dealing with earlier and current violence exposure for all staff members meeting both young men and women in their work. This would give young adults the opportunity to take a position on what they have gone through and learn how to deal with it so they could get on with their lives.

## Conclusions

Violence victimization in young men and women is not a single experience. Findings underline the importance of early interventions. Exposure to violence before 15 years of age is strongly associated with exposure to emotional, physical, and sexual abuse during the past 12 months in both young men and women, even after adjusting for sociodemographic factors and health risk behaviors such as hazardous drinking patterns, drug use, and daily smoking. It is time to consider routine questions on violence exposure for all staff meeting both young men and women in medical and social settings.

## Competing interests

The authors declare that they have no competing interests.

## Authors’ contributions

All authors read and approved the final manuscript. HB had the primary responsibility for acquisition, analysis, and interpretation of the data and writing the manuscript. UH participated in study design, analysis, and interpreting the study results, revised the manuscript, and provided inputs in various drafts. NO contributed to the analysis and interpreting the data and study results. ID designed the study, participated in acquisition of the data, analysis, and interpreting the study results, revised the manuscript, and provided inputs in various drafts.

## Pre-publication history

The pre-publication history for this paper can be accessed here:

http://www.biomedcentral.com/1471-2458/14/715/prepub
